# Heterozygote Advantage for Fecundity

**DOI:** 10.1371/journal.pone.0000125

**Published:** 2006-12-27

**Authors:** Neil J. Gemmell, Jon Slate

**Affiliations:** 1 School of Biological Sciences, University of Canterbury, Christchurch, New Zealand; 2 Department of Animal and Plant Sciences, University of Sheffield, Sheffield, United Kingdom; University of Cape Town, South Africa

## Abstract

Heterozygote advantage, or overdominance, remains a popular and persuasive explanation for the maintenance of genetic variation in natural populations in the face of selection. However, despite being first proposed more than 80 years ago, there remain few examples that fit the criteria for heterozygote advantage, all of which are associated with disease resistance and are maintained only in the presence of disease or other gene-by-environment interaction. Here we report five new examples of heterozygote advantage, based around polymorphisms in the *BMP15* and *GDF9* genes that affect female fecundity in domesticated sheep and are not reliant on disease for their maintenance. Five separate mutations in these members of the transforming growth factor β (*TGFβ*) superfamily give phenotypes with fitness differentials characteristic of heterozygous advantage. In each case, one copy of the mutant allele increases ovulation rate, and ultimately litter size per ewe lambing, relative to the wildtype. However, homozygous ewes inheriting mutant alleles from both parents have impaired oocyte development and maturation, which results in small undeveloped ovaries and infertility. Using data collected over many years on ovulation rates, litter size, and lambing rates, we have calculated the equilibrium solution for each of these polymorphisms using standard population genetic theory. The predicted equilibrium frequencies obtained for these mutant alleles range from 0.11 to 0.23, which are amongst the highest yet reported for a polymorphism maintained by heterozygote advantage. These are amongst the most frequent and compelling examples of heterozygote advantage yet described and the first documented examples of heterozygote advantage that are not reliant on a disease interaction for their maintenance.

## Introduction

Heterozygote advantage, or overdominance, remains a popular and persuasive explanation for the maintenance of genetic variation in natural populations in the face of selection [1], [2]. However, despite being first proposed more than 80 years ago [3], [4] there remain only a small number of examples that fit the criteria for heterozygote advantage, all of which are associated with disease resistance and maintained only in the presence of disease or other gene-by-environment interaction (see supporting text and Table S1). In addition, it is hard to continue to portray heterozygote advantage as an important general concept in evolutionary biology when a single example is used repeatedly throughout the literature. Here we report a simple, but compelling new example of heterozygote advantage, based around polymorphisms in genes associated with fertility in sheep.

Several genes have recently been identified that affect female fecundity in domesticated sheep [5]–[7]. Specifically, mutations in the *BMP15* [6] and *GDF9* [7] genes have been shown to increase ovulation rate and ultimately litter size in multiple sheep breeds (Table 1 [Table pone-0000125-t002]). The *BMP15* fecundity alleles show an X-linked overdominance inheritance pattern with infertility in homozygous females, while the *GDF9* fecundity alleles have an autosomal overdominance inheritance pattern with infertility in homozygous females [5]–[7]. Surprisingly, *BMP15* and *GDF9* have never been explicitly referred to as characteristic of heterozygote advantage and genotype fitnesses have never been estimated at these genes.

**Table 1:**
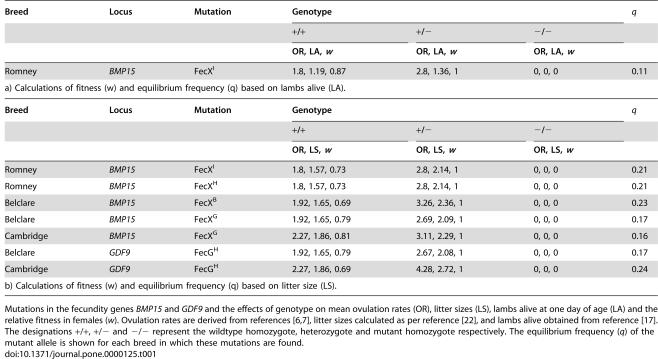
Fecundity genes exhibiting heterozygote advantage in sheep.

Breed	Locus	Mutation	Genotype			***q***
			+/+	+/−	−/−	
			OR, LA, *w*	OR, LA, *w*	OR, LA, *w*	
Romney	*BMP15*	FecX^I^	1.8, 1.19, 0.87	2.8, 1.36, 1	0, 0, 0	0.11
a) Calculations of fitness (w) and equilibrium frequency (q) based on lambs alive (LA).						

**Table pone-0000125-t002:**
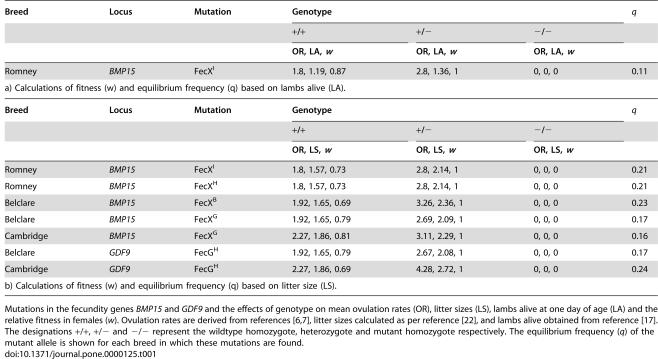


Breed	Locus	Mutation	Genotype			***q***
			+/+	+/−	−/−	
			OR, LS, *w*	OR, LS, *w*	OR, LS, *w*	
Romney	*BMP15*	FecX^I^	1.8, 1.57, 0.73	2.8, 2.14, 1	0, 0, 0	0.21
Romney	*BMP15*	FecX^H^	1.8, 1.57, 0.73	2.8, 2.14, 1	0, 0, 0	0.21
Belclare	*BMP15*	FecX^B^	1.92, 1.65, 0.69	3.26, 2.36, 1	0, 0, 0	0.23
Belclare	*BMP15*	FecX^G^	1.92, 1.65, 0.79	2.69, 2.09, 1	0, 0, 0	0.17
Cambridge	*BMP15*	FecX^G^	2.27, 1.86, 0.81	3.11, 2.29, 1	0, 0, 0	0.16
Belclare	*GDF9*	FecG^H^	1.92, 1.65, 0.79	2.67, 2.08, 1	0, 0, 0	0.17
Cambridge	*GDF9*	FecG^H^	2.27, 1.86, 0.69	4.28, 2.72, 1	0, 0, 0	0.24
b) Calculations of fitness (w) and equilibrium frequency (q) based on litter size (LS).						

Mutations in the fecundity genes *BMP15* and *GDF9* and the effects of genotype on mean ovulation rates (OR), litter sizes (LS), lambs alive at one day of age (LA) and the relative fitness in females (*w*). Ovulation rates are derived from references [6], [7], litter sizes calculated as per reference [22], and lambs alive obtained from reference [17]. The designations +/+, +/− and −/− represent the wildtype homozygote, heterozygote and mutant homozygote respectively. The equilibrium frequency (*q*) of the mutant allele is shown for each breed in which these mutations are found.

**Figure 1:**
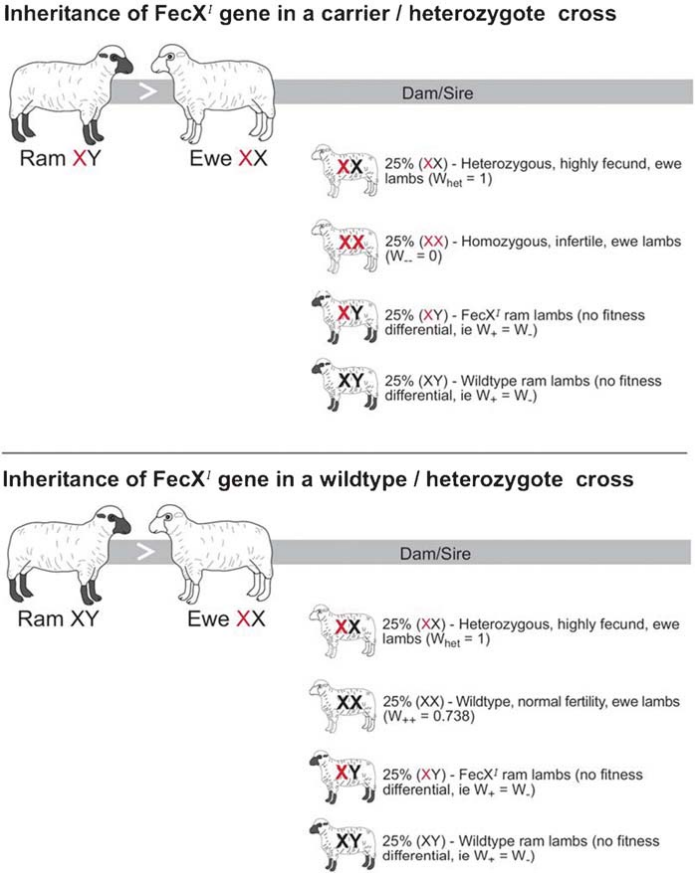
Genotypes and the fitness differentiations (w) for the Inverdale mutation (FecXI).

Four separate mutations (Inverdale, Hanna, Belclare and Galway) in the X-linked *BMP15* gene (Table 1 [Table pone-0000125-t002]) have been described [5] that give rise to phenotypes with fitness differentials characteristic of heterozygous advantage (Figure 1). One copy of the Inverdale (*FecX^I^*), Hanna (*FecX^H^*), Belclare (*FECX^B^*) or Galway (*FECX^G^*) allele increases ovulation rate and ultimately litter size by about 0.6 lambs per ewe lambing compared to the wildtype [5]–[7]. However, homozygous ewes inheriting mutant alleles from both parents have small undeveloped ovaries and are infertile [6]–[8].

Mechanistically, the genetic basis of this heterozygote advantage is well understood. The *BMP15* gene encodes bone morphogenic protein 15 (also known as growth differentiation factor 9B, GDF9B), which is a member of the transforming growth factor β (*TGFβ*) superfamily [6]. *BMP15* acts through a cascade of other proteins (the SMAD pathway) that are responsible for a huge diversity of cellular behaviours, including oocyte development and maturation [6], [8], [9]. Without *BMP15*, oocytes continue to grow in the absence of granulosa cell proliferation until they are unable to be supported by the residual granulosa cells, whereupon they degenerate [6]. Curiously, although a lack of *BMP15* blocks follicular growth in homozygotes, inactivation of only one copy of *BMP15* increases ovulation rate [6], [8], conferring a fecundity advantage to the heterozygote (Table 1 [Table pone-0000125-t002], Figure 1).

A similar pattern of heterozygote advantage is observed in another closely related member of theTGFβ superfamily *GDF9* [10]. Like *BMP15*, the autosomal *GDF9* has an essential role in controlling follicular growth via its influence on granulosa cell function [7], [10]. An absence of *GDF9* also blocks follicular growth in homozygotes, resulting in sterility, while inactivation of only one copy of *GDF9* again increases ovulation rate [7], conferring a fecundity advantage to the heterozygote (Table 1 [Table pone-0000125-t002]).

Despite being very compelling examples of heterozygote advantage, the overdominant nature of these fertility polymorphisms is not widely recognised. One of the hurdles to these becoming a more widely known example of heterozygote advantage is the absence of data on the equilibrium solution for each of these polymorphisms and knowledge of their frequencies in a natural situation (see supporting text). However, using standard population genetic theory [11] the equilibrium solution can be readily calculated from data collected over many years on ovulation rates, litter size and lambing rates [5]–[7]. Here we report the predicted fitness and equilibrium solution for each of these fecundity polymorphisms and show that these are expected to be amongst the most frequent and compelling examples of heterozygote advantage yet described.

## Results and Discussion

The equilibrium solution for each fecundity polymorphism was calculated from data collected on ovulation rates, litter size, and lambing rates as described below. For the Inverdale mutation (*FecX^I^*) the estimated relative fitness of ewes (*w*), based on lambs alive at one day of age, of the homozygote wildtype (*++*), heterozygote (*+/−*) and mutant homozygote (*−/−*) are 0.87, 1 and 0 respectively, leading to a predicted equilibrium frequency for the *FecX^I^* allele (*q*) of 0.11 (Table 1a). Other mutations in *BMP15* and *GDF9* show similar relative fitnesses and equilibrium frequencies for the more limited litter size data (Table 1b), which are collectively amongst the highest yet described for a polymorphism maintained by heterozygote advantage. For comparison, the equilibrium frequency of the recessive sickle cell allele (Hb^S^) rarely exceeds 0.15 [12].

Heterozygote advantage as a concept holds considerable sway in evolutionary biology, but only a handful of examples have withstood close scrutiny (reviewed in Table S1). Of the 21 examples reported that invoke heterozygote advantage as an explanation for the maintenance of polymorphism, compelling evidence for heterozygote advantage is lacking in nearly every instance (Table S1). The following information is required to unequivocally demonstrate heterozygote advantage: (i) the gene and mutant alleles under selection must be known; (ii) the relative fitness of each genotype must be known (with heterozygotes exhibiting the greatest relative fitness); (iii) the mechanism of selection must be understood i.e. we need to known why heterozygotes are fitter than homozygotes.

Prior to the discovery of the sheep fecundity genes described here, perhaps only the classic example of heterozygote advantage, sickle cell anaemia, met these requirements. In African and Asian human populations where malaria is prevalent, fatalities in homozygotes for the sickle cell trait (Hb^SS^) are offset by the survival advantage of heterozygotes bearing one copy of the sickle cell allele (Hb^S+^) over the wildtype homozygotes (Hb^++^), when challenged with malaria [1], [12]. Sickle cell anaemia is an excellent example of heterozygote advantage, but it has two principal shortcomings. First, the advantage to heterozygotes is conditional, only operating where malaria is prevalent [1]. Second, the heterozygote advantage arises because of the super-position of two opposite directional selection pressures (malaria and sickle-cell anaemia), so it is not really a true case of heterozygote advantage arising from the heterozygote having superior fitness for a specific trait.

There are numerous other putative examples of heterozygote advantage that evoke contemporary and/or historical resistance to infectious disease to explain the relatively high frequency of otherwise deleterious alleles. Examples include the cystic fibrosis-causing alleles at the *CFTR* gene, the Gaucher Disease-causing allele at *GBA*, deafness-causing alleles at *GJB2* and polymorphism at the major histocompatability locus *MHC* (Table S1). However, in the majority of these cases genotype fitnesses remain undetermined, and very often the infectious disease driving selection is unknown. This missing information makes it difficult to distinguish between heterozygote advantage and alternative explanations including other forms of balancing selection, such as negative frequency dependence or spatially/temporally variable directional selection [13].

The fertility polymorphisms documented in *BMP15* and *GDF9* represent one of the best examples of heterozygote advantage yet described, with the genetic basis, fitness differentials and the mechanism of selection well understood [5]–[7]. Crucially, in stark contrast to sickle cell anaemia, no external factor, such as disease [2], needs to be invoked to understand the fitness differentials among genotypes for these polymorphisms.

There are of course still substantial issues unresolved for these polymorphisms too. Among the most important of these issues is the frequency of the polymorphism in the wild. At equilibrium our theoretical expectation, based on data from domesticated sheep, is that fertility alleles such as *FecX^I^* will occur at a frequency of 0.11–0.23 (Table 1 [Table pone-0000125-t002]). The obvious extension of this work then is to screen wild populations of sheep for these mutations to determine if they are present and if the frequencies match expectations. To date such an investigation has not been undertaken widely. One study of a small population of Soay sheep on the remote Islands of St Kilda found no evidence for any of the *BMP15* fertility polymorphisms described in Table 1 [Table pone-0000125-t002] (Gratten pers. comm.). However since this is an isolated population, subject to regular population crashes [14], the absence of polymorphism in *BMP15* in this population may not be overly surprising and further studies in other wild sheep populations are warranted.

Heterozygote advantage is commonly assumed to slow the rate of loss of genetic diversity by genetic drift. However, Robertson [15] described scenarios where it can accelerate loss of diversity by drift, and provided solutions to the relative rate that heterozygote advantage accelerates or retards loss of diversity for populations of finite size. Extrapolating from Table 1 [Table pone-0000125-t002] of Robertson [15], and assuming that s_1_ = 0.125, s_2_ = 0.5 because selection is only in one sex, and q = 0.20, then heterozygote advantage at the loci described here would retard the effects of genetic drift 100-fold in a population with effective population size (N_e_) 176 (retardation of loss would be greater in larger populations). In other words, the polymorphism is likely to be maintained in moderate or large sized populations.

It may well transpire that the fertility polymorphisms described here are only relatively common in domesticated sheep as a consequence of selective breeding and that they do not actually confer an appreciable fitness advantage to heterozygotes in the wild, due perhaps to the existence of other genes under stronger selection that are in linkage or have epistatic effects on these fertility polymorphisms. One prominent example of a mutation maintained at high frequency by human mediated selection for the heterozygote is the Halothane mutation in pigs [16]. The Halothane mutation occurs as a result of a missense mutation in a calcium release gene, the ryanodine receptor gene (*RYR1*), expressed in muscle. Heterozygous carriers show a higher lean content than homozygous wild type individuals, whereas the mutant homozygotes are highly susceptible to malignant hyperthermia [16]. Selection for leanness in domesticated pigs created an overdominant situation maintaining the mutation at high frequency until a DNA based diagnostic became available that enabled the targeted culling of individuals bearing the Halothane mutation [16].

It remains possible that the fertility polymorphisms in *BMP15* and *GDF9* are also maintained through selective breeding, but these mutations differ significantly from the Halothane mutation in that the heterozygotes clearly have a natural advantage in terms of fecundity over the wild type, with little demonstrable decrease in other fitness parameters for the heterozygote reported to date [5]–[7], [17]. However, an obvious possibility is that there is a trade off between birthing many lambs and their survival to weaning, particularly when environmental conditions are unfavourable. To explore this further we have examined data on the mean frequency of single and multiple births for carriers of the Inverdale mutation (FecX^I^) and for wildtype Romney sheep [18]. We have then estimated the relative fitness of each genotype using data on average survival rates to weaning, for lambs for each birth category, across environmental conditions that range from intensive farming to harsh hill country (Table 2 [Table pone-0000125-t004]) [18]. Due to an absence of data on the frequency of different litter sizes under different environmental conditions, we have made the conservative assumption that this is unaffected by environmental conditions.

**Table 2:**
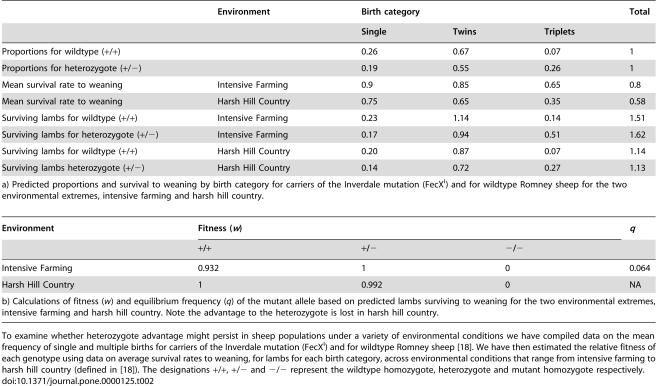
Table 2. Heterozygote advantage for fecundity in sheep under two different environment conditions.

	Environment	Birth category			Total
		Single	Twins	Triplets	
Proportions for wildtype (+/+)		0.26	0.67	0.07	1
Proportions for heterozygote (+/−)		0.19	0.55	0.26	1
Mean survival rate to weaning	Intensive Farming	0.9	0.85	0.65	0.8
Mean survival rate to weaning	Harsh Hill Country	0.75	0.65	0.35	0.58
Surviving lambs for wildtype (+/+)	Intensive Farming	0.23	1.14	0.14	1.51
Surviving lambs for heterozygote (+/−)	Intensive Farming	0.17	0.94	0.51	1.62
Surviving lambs for wildtype (+/+)	Harsh Hill Country	0.20	0.87	0.07	1.14
Surviving lambs heterozygote (+/−)	Harsh Hill Country	0.14	0.72	0.27	1.13
a) Predicted proportions and survival to weaning by birth category for carriers of the Inverdale mutation (FecX^I^) and for wildtype Romney sheep for the two environmental extremes, intensive farming and harsh hill country.					

**Table pone-0000125-t004:**
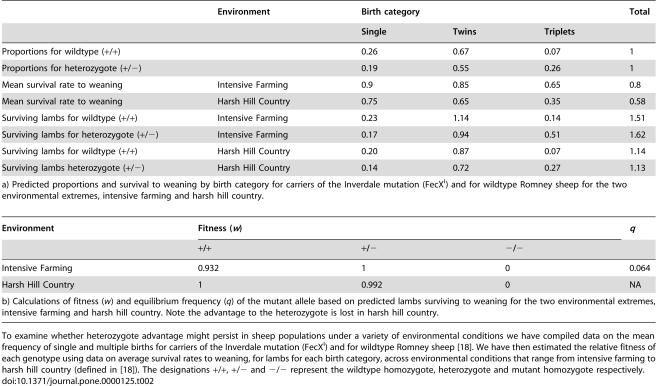


Environment	Fitness (*w*)			*q*
	+/+	+/−	−/−	
Intensive Farming	0.932	1	0	0.064
Harsh Hill Country	1	0.992	0	NA
b) Calculations of fitness (*w*) and equilibrium frequency (*q*) of the mutant allele based on predicted lambs surviving to weaning for the two environmental extremes, intensive farming and harsh hill country. Note the advantage to the heterozygote is lost in harsh hill country.				

To examine whether heterozygote advantage might persist in sheep populations under a variety of environmental conditions we have compiled data on the mean frequency of single and multiple births for carriers of the Inverdale mutation (FecX^I^) and for wildtype Romney sheep [18]. We have then estimated the relative fitness of each genotype using data on average survival rates to weaning, for lambs for each birth category, across environmental conditions that range from intensive farming to harsh hill country (defined in [18]). The designations +/+, +/− and −/− represent the wildtype homozygote, heterozygote and mutant homozygote respectively.

We found that lamb survival to weaning does decrease with increasing litter size [17], [18], but that the advantage to heterozygotes seems to hold across a wide variety of environmental conditions (Table 2 [Table pone-0000125-t004]), even with our rather simplistic assumption that the frequency of births by category is unaffected by environment. Under intensive farming the selection coefficient (*s*) in favour of the heterozygote is 0.068, whilst in harsh conditions the wildtype is favoured slightly with *s* of 0.008 (Table 2 [Table pone-0000125-t004]). These calculations suggest that the heterozygote advantage for fecundity conferred by *BMP15* has some dependence on environmental conditions. Under benign conditions (e.g. intensive farming, easy and hard hill country), the heterozygous advantage would be promoted and equilibrium might be reached, but under harsh conditions (e.g. harsh hill country), lamb survival for multiple births drops [18] and the advantage to heterozygotes is apparently lost (Table 2 [Table pone-0000125-t004]).

Recalculating the equilibrium solution for the mutant allele under each of these environmental scenarios results in an expected equilibrium frequency, *q*, of no greater than 0.064 predicted for intensive farming, the most benign situation (Table 2 [Table pone-0000125-t004]). This is an important result because based on the calculations of Robertson [15] a value of *q* below 0.2 will usually result in heterozygous advantage actually accelerating the effects of drift unless N_e_ is very large, making a stable equilibrium increasingly unlikely as *q* becomes smaller.

Overall our calculations suggest that a stable equilibrium for these fecundity alleles might only be expected in populations of large size (Ne>400) [15] under favourable or predominantly favourable environmental conditions. However, it remains possible that the heterozygous advantage could be maintained in natural populations even under harsh conditions if females modify the number of lambs they produce in response to environmental conditions, which a range of studies suggest they will [19]–[21]. This is a factor that we have been unable to incorporate in our calculations due to an absence of condition specific rates of single, twin and other multiple births. However, if heterozygous carriers and wildtype ewes responded in the same way to the harsher environments, producing fewer multiple births, it may transpire that the heterozygote may still have superior fitness across all environmental conditions.

The possibility remains that the alleles that confer this heterozygous advantage may be maintained in wild populations and further research is warranted. Investigations of the patterns of genomic diversity adjacent to each of these loci would provide insights into any obvious pattern of selection around these genes. In addition, such a study might enable the estimation of the age of these polymorphisms, which we would predict would be old, if indeed they are maintained by overdominance in the wild. While there is still much to be determined about the maintenance of these new examples of heterozygote advantage in the wild, the overdominant effect of these mutations under domestication is incontrovertible, and provides a useful complement to the perennial example of heterozygote advantage based upon the sickle cell [1].

## Materials and Methods

The relative genotype fitnesses in females (*w*) and the equilibrium frequency (*q*) of the mutant allele were calculated for each of the *BMP15* and *GDF9* polymorphisms found in each breed of sheep (Table 1 [Table pone-0000125-t002]). For these calculations we assume that litter size (LS), is an accurate measure of female reproductive fitness. To calculate litter size (LS), we used reports of ovulation rate (OR) [6], [7], the standard measure of fecundity in this system, which we converted using the quadratic equation:
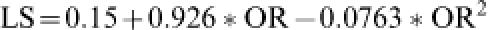

[22]

Subsequently, where data were available, we have adjusted for neo-natal mortality, to give an estimate of the lambs alive at one day of age per ewe lambing (LA), which incorporates barrenness, litter size and lamb survival into a single measure [17].

*BMP15* (*GDF9B*) is X-linked therefore males have only one copy of the gene and there is no evidence of a fitness difference between alleles in males [5]. Therefore, assuming that wild type females have relative fitness 1-s, heterozygous females have relative fitness 1, and female mutant homozygotes have relative fitness 1-t, and that male carriers have fitness equal to male wild types, the equilibrium frequency of the mutant allele is given by *q* = s/(s+t) [11]. Note that since FecX^B^ and FecX^G^ both segregate in Belclare sheep a stable equilibrium cannot be attained when both mutants are present [6], [7]. Therefore, for simplicity, the reported equilibrium frequency is based on the assumption that only the focal mutant and the wild type segregate.

*GDF9* maps to sheep chromosome 5 and there is no evidence of a fitness difference between genotypes in males [5]. Again, we assumed that the three genotypes have fitness 1-s, 1 and 1-t in females as above, but in this case the three male genotypes have relative fitness 1, halving the selection coefficient against the two homozygous genotypes. The equilibrium frequency of the mutant allele is given by *q* = 0.5s/(0.5s+0.5t) = s/(s+t) [11].

## Supporting Information

Table S1 A review of genes proposed to exhibit heterozygote advantage.
(0.12 MB DOC)
