# Supplementary material for: Heterozygote Advantage for Fecundity
Source: PLoS One. 2006 Dec 27;1(1):e125. doi: 10.1371/journal.pone.0000125 (PMC1762409; doi:10.1371/journal.pone.0000125)
Supplement: Table S1 — A review of genes proposed to exhibit heterozygote advantage. (0.12 MB DOC) [file pone.0000125.s001.doc]

Supporting Online Material for: Heterozygote Advantage for Fecundity

Neil J. Gemmell^1*^ and Jon Slate^2^

*1. School of Biological Sciences, University of Canterbury, Private Bag 4800, Christchurch, NZ.*

*2. Department of Animal and Plant Sciences, University of Sheffield, Sheffield, UK, S10 2TN*

**Corresponding author: e-mail: neil.gemmell@canterbury.ac.nz*

# Table S1: A review of genes proposed to exhibit heterozygote advantage

There are many putative examples of genes where heterozygote advantage acts to maintain genetic variation. However, in nearly every instance, compelling evidence for heterozygote advantage has not yet been obtained. In our view the following pieces of information are required to unequivocally demonstrate heterozygote advantage: (i) the gene and mutant alleles under selection must be known; (ii) the relative fitness of each genotype must be measured (with heterozygotes exhibiting greatest relative fitness); (iii) the mechanism of selection must be understood i.e. the reason why heterozygotes are fitter than homozygotes. Below, we have compiled a comprehensive, but non-exhaustive, list of genes that may have been subject to historical or contemporary heterozygote advantage. Alternative explanations, other than heterozygote advantage cannot be dismissed for most examples.

| **Species** | ***Locus*** | **Evidence for heterozygote advantage** | **Alternative Explanation** |
| --- | --- | --- | --- |
| *H. sapiens* | *HBB* | S allele heterozygotes have increased resistance to malaria; homozygotes have sickle cell anaemia [1]. | None, the textbook example of heterozygote advantage. |
|  | *CFTR* | Ancestral heterozygotes proposed to have increased resistance to cholera [2]; homozygotes have cystic fibrosis. | Cholera reached Europe too late for *CFTR* mutant alleles to have reached the observed frequencies. Alternative explanations include heterozygous resistance to typhoid [3] and asthma [4], but none demonstrated unequivocally in humans. |
|  | *HBA α-thalassemia* | Heterozygotes have increased resistance to malaria; homozygotes have α-thalassemia [5]. | Disease is relatively mild and homozygotes are more resistant than heterozygotes; directional selection cannot be excluded [6]. In addition, α-thalassemia alleles are also associated with other infectious diseases [6] e.g. Thalassemic children in Vanuatu more susceptible to infection with *Plasmodium vivax*. Subsequent immune response renders them relatively resistant to infection with *P. falciparum*. i.e. disease resistance complicated by age * genotype interaction. |
|  | *HBB β-thalassemia* | Heterozygotes have increased resistance to malaria; homozygotes have β-thalassemia . | No evidence that thalassemic red cells show enhanced resistance to invasion by *Plasmodium falciparum*. Relative fitnesses of Hb-β thalassemia allele-bearing genotypes unknown. Some alleles (e.g. *HBB* C) appear to be subject to directional selection [7-9]. |
|  | *HLA (MHC)* | Heterozygosity at the *HLA* (*MHC*), the highly polymorphic loci that control immunological recognition of pathogens, is suspected to confer a selective advantage by enhancing resistance to infectious diseases (the "heterozygote advantage" hypothesis) [10]. Evidence in some non-human systems also suggest that heterozygosity at the *HLA* may confer reproductive advantages [11]. | Heterozygote advantage on its own is insufficient to explain the high population diversity of the *HLA* (MHC) region [12]. |
|  | *TDS* | The high frequency of Tay-Sachs disease in Ashkenazi Jewish population has been suggested to be a consequence of heterozygote advantage, with heterozygotes possessing increased resistance to diseases, such as tuberculosis [13]. | Proportionally the grandparents of Tay-Sachs disease carriers died from the same causes as grandparents of non carriers so the high frequency of the TSD gene in Ashkenazim is probably caused by a combination of founder effect, genetic drift, and differential immigration patterns [14]. |
|  | *TDS and GD* | The presence of four lysosomal storage diseases, particularly Tay-Sachs and Gaucher disease type I, at increased frequency in the Ashkenazi Jewish population is suggestive of heterozygote advantage operating on fat (sphingolipid) storage processes [15]. | The geographic distribution of these disease mutations in the Ashkenazi Jewish population supports genetic drift, rather than selection, as the mechanism of unusually high frequency of conditions [16]. |
|  | *G6PD* | X-linked gene. Female homozygous for *G6PD* mutant alleles have nonspherocytic haemolytic anaemia. Heterozygous females and hemizygous males proposed to be malaria resistant [17,18]. | Nonspherocytic haemolytic anaemia is relatively benign. Mutant alleles may have reached high frequencies due to positive selection, not heterozygote advantage [19,20]}. |
|  | *PRNP* | Among the Fore women of Papua New Guinea heterozygotes appear to have increased resistance to kuru [21,22]. | Analysis limited to 30 women. Evidence for balancing selection disputed [23]. |
|  | *GJB2* | Homozygotes have deafness; heterozygotes proposed to have increased cell survival and thicker epidermis, thereby increasing resistance to infection by pathogens [24,25]. | Evidence for heterozygote advantage speculative. Infectious agent and relative genotype fitnesses unknown. |
|  | *GBA* | Homozygotes have Gaucher disease, high frequency of disease alleles in Ashkenazi Jews taken as evidence of heterozygote advantage [26]. | No association between genotype and any trait other than Gaucher disease. Therefore, no direct evidence of heterozygote advantage. Population genetic models suggest high frequency of deleterious recessive alleles in Ashkenazi Jews can be explained by founder effects; i.e. balancing selection is not necessary to explain contemporary allele frequencies [27]. |
|  | *MTHFR* | Heterozygous females with neural tube defects less likely to abort than homozygous females with neural tube defects, heterozygous males less likely to have neural tube defects than homozygotes [28]. | No estimate of relative fitness of different genotypes. Other studies have found no association between neural tube defects and *MTHFR*. No departure from Hardy-Weinberg equilibrium in cohort of 80 year olds [29]. |
|  | *PAH* | Homozygous individuals have phenylketonuria (*PKU*); heterozygotes proposed to be resistant to ochratoxin A, a mycotoxin produced by aspergillus and penicillium species that infest grains [30]. | Evidence for heterozygous advantage, including relative genotype fitnesses and mechanism of selection is unknown [31]. |
|  | *CCR5* | Individuals who are homozygous for *CCR5* mutant alleles show increased resistance to HIV infection. Heterozygotes show slower progression to AIDS than wild type homozygotes [32,33]. Despite recent HIV-driven directional selection, the high frequency of mutant *CCR5* alleles, is suggestive of historic heterozygote advantage acting on *CCR5* [34]. | Mechanism of historic heterozygote advantage unknown. Resistance to either plague [35] or smallpox are possible explanations [36]. |
|  | *PTC* | Homozygotes for mutant alleles cannot perceive bitter tastes. High frequency of non-taster alleles in many populations and molecular evolution analysis suggestive of balancing selection [37]. | Heterozygote advantage and other forms of balancing selection cannot be distinguished. No known advantage of heterozygous genotype. Relative genotype fitnesses unknown. |
|  | *PCDHA* | Gene involved in synaptic complexity. Molecular evolution analysis suggestive of balancing selection [38]. | Heterozygote advantage and other forms of balancing selection cannot be distinguished. No known advantage of heterozygous genotype. Relative genotype fitnesses unknown. |
|  | *MEFV* | Individuals homozygous for *MEFV* mutations have Familial Mediterranean Fever (FMF). Proposed that heterozygotes have reduced susceptibility to tuberculosis [39]. | No documented association between *MEFV* and tuberculosis susceptibility. Relative genotype fitnesses remain unreported. Evidence of positive selection rather than balancing selection [40]. |
| *Rattus norvegicus* | *RW* | Heterozygotes for an *RW* mutant are resistant to the pesticide, warfarin. Homozygotes have a greater vitamin K requirement and are therefore less viable [41]. | Experimental [42] and molecular evolution [43] evidence supports directional selection rather than heterozygote advantage. |
| *Columba livia* | *TF* | Female pigeons heterozygous for the two common alleles, *TFa* and *TFb*, had lower microbial infection and higher egg hatching rates than either homozygote [44]. | None, but this remains a relatively unexplored system. This apparent example of heterozygote advantage is not widely known, cited just three times since 2000. |
| *Colias meadii* | *PGI* | Data on male mating success of common *C. meadii* *PGI* genotypes in steppe and tundra show heterozygote advantage in both habitat types, with shifts in relative homozygote disadvantage between habitats which are consistent with the observed frequency differences [45]. | Directional selection may be acting against the *PGI* alleles at local scales as a consequence of microclimatic thermal variables [45]. |
| *Culex pipiens* | *Ace.1*  *Ester* | At both loci, dominant alleles that are resistant to pesticides are at high frequency in Southern France. In areas where pesticide treatment is absent, resistance alleles are at lower frequency [46]. | Genetic variation is maintained by mutation-selection balance rather than heterozygote advantage per se. Then relative fitness of the different genotypes varies with season and location. Thus apparent heterozygote advantage relies on frequency-dependent selection and a fluctuating environment. |

**Supporting References**

1. Allison AC (1954) Protection afforded by sickle-cell trait against subtertian malarial infection. Br Med J 1: 290-294.

2. Gabriel SE, Brigman KN, Koller BH, Boucher RC, Stutts MJ (1994) Cystic-fibrosis heterozygote resistance to cholera-toxin in the cystic-fibrosis mouse model. Science 266: 107-109.

3. Pier GB, Grout M, Zaidi T, Meluleni G, Mueschenborn SS, et al. (1998) *Salmonella typhi* uses CFTR to enter intestinal epithelial cells. Nature 393: 79-82.

4. Schroeder SA, Gaughan DM, Swift M (1995) Protection against Bronchial-Asthma by Cftr Delta-F508 Mutation - a Heterozygote Advantage in Cystic-Fibrosis. Nat Med 1: 703-705.

5. Flint J, Hill AVS, Bowden DK, Oppenheimer SJ, Sill PR, et al. (1986) High-frequencies of alpha-thalassemia are the result of natural-selection by malaria. Nature 321: 744-750.

6. Allen SJ, O'Donnell A, Alexander NDE, Alpers MP, Peto TEA, et al. (1997) alpha +-Thalassemia protects children against disease caused by other infections as well as malaria. Proc Nat Acad Sci U S A 94: 14736-14741.

7. Hedrick P (2004) Estimation of relative fitnesses from relative risk data and the predicted future of haemoglobin alleles S and C. J Evol Biol 17: 221-224.

8. Modiano D, Luoni G, Sirima BS, Simpore J, Verra F, et al. (2001) Haemoglobin C protects against clinical *Plasmodium falciparum* malaria. Nature 414: 305-308.

9. Wood ET, Stover DA, Slatkin M, Nachman MW, Hammer MF (2005) The [beta]-globin recombinational hotspot reduces the effects of strong selection around HbC, a recently arisen mutation providing resistance to malaria. Am J Hum Genet 77: 637-642.

10. Penn DJ, Damjanovich K, Potts WK (2002) MHC heterozygosity confers a selective advantage against multiple-strain infections. Proc Natl Acad Sci U S A 99: 11260-11264.

11. Sauermann U, Nurnberg P, Bercovitch FB, Berard JD, Trefilov A, et al. (2001) Increased reproductive success of MHC class II heterozygous males among free-ranging rhesus macaques. Hum Genet 108: 249-254.

12. De Boer RJ, Borghans JA, van Boven M, Kesmir C, Weissing FJ (2004) Heterozygote advantage fails to explain the high degree of polymorphism of the MHC. Immunogenetics 55: 725-731.

13. Yokoyama S (1979) Role of genetic drift in the high frequency of Tay-Sachs disease among Ashkenazic Jews. Ann Hum Genet 43: 133-136.

14. Spyropoulos B, Moens PB, Davidson J, Lowden JA (1981) Heterozygote advantage in Tay-Sachs carriers? Am J Hum Genet 33: 375-380.

15. Peleg L, Frisch A, Goldman B, Karpaty M, Narinsky R, et al. (1998) Lower frequency of Gaucher disease carriers among Tay-Sachs disease carriers. Eur J Hum Genet 6: 185-186.

16. Risch N, Tang H, Katzenstein H, Ekstein J (2003) Geographic distribution of disease mutations in the Ashkenazi Jewish population supports genetic drift over selection. Am J Hum Genet 72: 812-822.

17. Luzatto L, FA U, Reddy S (1969) Glucose-6-phosphate dehydorgenase deficient red cells: resistant to infection by malarial parasites. Science 164: 839-842.

18. Ruwende C, Khoo SC, Snow RW, Yates SNR, Kwiatkowski D, et al. (1995) Natural selection of hemi- and heterozygotes for G6PD deficiency in Africa by resistance to severe malaria. Nature 376: 246-249.

19. Saunders MA, Hammer MF, Nachman MW (2002) Nucleotide variability at G6pd and the signature of malarial selection in humans. Genetics 162: 1849-1861.

20. Tishkoff SA, Varkonyi R, Cahinhinan N, Abbes S, Argyropoulos G, et al. (2001) Haplotype diversity and linkage disequilibrium at human G6PD: Recent origin of alleles that confer malarial resistance. Science 293: 455-462.

21. Hedrick PW (2003) A heterozygote advantage. Science 302: 57-57.

22. Mead S, Stumpf MPH, Whitfield J, Beck JA, Poulter M, et al. (2003) Balancing selection at the prion protein gene consistent with prehistoric kuru like epidemics. Science 300: 640-643.

23. Kreitman M, Di Rienzo A (2004) Balancing claims for balancing selection. Trends Genet 20: 300-304.

24. Common JEA, Di WL, Davies D, Kelsell DP (2004) Further evidence for heterozygote advantage of GJB2 deafness mutations: a link with cell survival. J Med Genet 41: 573-575.

25. Meyer CG, Amedofu GK, Brandner JM, Pohland D, Timmann C, et al. (2002) Selection for deafness? Nat Med 8: 1332-1333.

26. Boas FE (2000) Linkage to Gaucher mutations in the Ashkenazi population: Effect of drift on decay of linkage disequilibrium and evidence for heterozygote selection. Blood Cells Mol Dis 26: 348-359.

27. Slatkin M (2004) A population-genetic test of founder effects and implications for Ashkenazi Jewish diseases. Am J Hum Genet 75: 282-293.

28. Schwartz CE, Hunter AGW, Holmes LB, Guttormsen SA, Tackels DC, et al. (1997) The methylenetetrahydrofolate reductase (MTHFR) C677T polymorphism: An example of heterozygote advantage by natural selection. Am J Hum Genet 61: A211-A211.

29. Visscher PM, Tynan M, Whiteman MC, Pattie A, White I, et al. (2003) Lack of association between polymorphisms in angiotensin-converting-enzyme and methylenetetrahydrofolate reductase genes and normal cognitive ageing in humans. Neurosci Lett 347: 175-178.

30. Woolf LI (1986) The heterozygote advantage in phenylketonuria. Am J Hum Genet 38: 773-775.

31. Krawczak M, Zschocke J (2003) A role for overdominant selection in phenylketonuria? Evidence from molecular data. Hum Mutat 21: 394-397.

32. Dean M, Carrington M, Winkler C, Huttley GA, Smith MW, et al. (1996) Genetic restriction of HIV-1 infection and progression to AIDS by a deletion allele of the CKR5 structural gene. Science 273: 1856-1862.

33. Liu R, Paxton WA, Choe S, Ceradini D, Martin SR, et al. (1996) Homozygous defect in HIV-1 coreceptor accounts for resistance of some multiply-exposed individuals to HIV-1 infection. Cell 86: 367-377.

34. Bamshad MJ, Mummidi S, Gonzalez E, Ahuja SS, Dunn DM, et al. (2002) A strong signature of balancing selection in the 5 ' cis-regulatory region of CCR5. Proc Nat Acad Sci U S A 99: 10539-10544.

35. Duncan SR, Scott S, Duncan CJ (2005) Reappraisal of the historical selective pressures for the CCR5-Delta 32 mutation. J Med Genet 42: 205-208.

36. Galvani AP, Slatkin M (2003) Evaluating plague and smallpox as historical selective pressures for the CCR5-Delta 32 HIV-resistance allele. Proc Natl Acad Sci U S A 100: 15276-15279.

37. Wooding S, Kim UK, Bamshad MJ, Larsen J, Jorde LB, et al. (2004) Natural selection and molecular evolution in PTC, a bitter-taste receptor gene. Am J Hum Genet 74: 637-646.

38. Noonan JP, Li J, Nguyen L, Caoile C, Dickson M, et al. (2003) Extensive linkage disequilibrium, a common 16.7-kilobase deletion, and evidence of balancing selection in the human protocadherin alpha cluster. Am J Hum Genet 72: 621-635.

39. Torosyan Y, Aksentijevich I, Sarkisian T, Astvatsatryan V, Ayvazyan A, et al. (1999) A population-based survey reveals an extremely high FMF carrier frequency in Armenia, suggesting heterozygote advantage. Am J Hum Genet 65: A400-A400.

40. Schaner P, Richards N, Wadhwa A, Aksentijevich I, Kastner D, et al. (2001) Episodic evolution of pyrin in primates: human mutations recapitulate ancestral amino acid states. Nat Genet 27: 318-321.

41. Greaves JH, Redfern R, Ayres PB, Gill JE (1977) Warfarin resistance - balanced polymorphism in Norway rat. Genet Res 30: 257-263.

42. Partridge GG (1979) Relative fitness of genotypes in a population of *Rattus norvegicus* polymorphic for warfarin resistance. Heredity 43: 239-246.

43. Kohn MH, Pelz HJ, Wayne RK (2000) Natural selection mapping of the warfarin-resistance gene. Proc Natl Acad Sci U S A 97: 7911-7915.

44. Frelinger JA (1972) Maintenance of transferrin polymorphism in pigeons. Proc Nat Acad Sci U S A 69: 326-329.

45. Watt WB, Wheat CW, Meyer EH, Martin J-F (2003) Adaptation at specific loci. VII. Natural selection, dispersal and the diversity of molecular-functional variation patterns among butterfly species complexes (Colias: Lepidoptera, Pieridae). Mol Ecol 12: 1265-1275.

46. Lenormand T, Bourguet D, Guillemaud T, Raymond M (1999) Tracking the evolution of insecticide resistance in the mosquito *Culex pipiens*. Nature 400: 861.
